# Distribution of polymeric nanoparticles in the eye: implications in ocular disease therapy

**DOI:** 10.1186/s12951-020-00745-9

**Published:** 2021-01-07

**Authors:** Sean Swetledge, Jangwook P. Jung, Renee Carter, Cristina Sabliov

**Affiliations:** 1 Department of Biological and Agricultural Engineering, Louisiana State University, Baton Rouge, LA 70803 USA; 2 Veterinary Clinical Sciences, Louisiana State University and LSU Veterinary Medicine, Skip Bertman Drive, Baton Rouge, LA 70803 USA; 3 Department of Biological and Agricultural Engineering, Louisiana State University and LSU Agricultural Center, Baton Rouge, LA 70803 USA

**Keywords:** Ocular biodistribution, Polymeric nanoparticle, Ocular drug delivery, Drug delivery system

## Abstract

Advantages of polymeric nanoparticles as drug delivery systems include controlled release, enhanced drug stability and bioavailability, and specific tissue targeting. Nanoparticle properties such as hydrophobicity, size, and charge, mucoadhesion, and surface ligands, as well as administration route and suspension media affect their ability to overcome ocular barriers and distribute in the eye, and must be carefully designed for specific target tissues and ocular diseases. This review seeks to discuss the available literature on the biodistribution of polymeric nanoparticles and discuss the effects of nanoparticle composition and administration method on their ocular penetration, distribution, elimination, toxicity, and efficacy, with potential impact on clinical applications. 
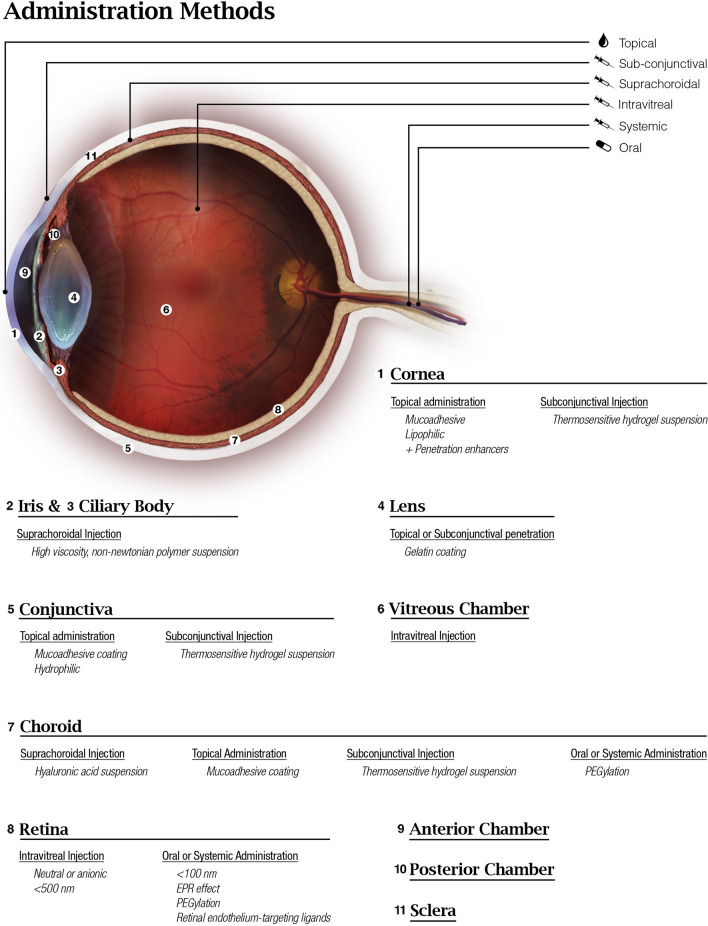

## Background

Over 1 billion people suffered from some form of visual impairment in 2015, of which an estimated 36 million individuals suffered from blindness [1]. Unfortunately, drug delivery to the inner eye is complicated by anatomical barriers to systemic and topical administration as well as complications of repeated intravitreal drug administration [2]. A potential strategy to improve drug delivery to the eye utilizes polymeric nanoparticles, which have tunable size and surface properties designed to ensure successful transit of the drug to the target tissue in the eye as well as potential for a controlled drug release profile, reducing the required treatment frequency. While several reviews have addressed the potential applications of polymeric nanoparticles in ocular delivery [3–7], no reviews to our knowledge specifically focus on the ocular biodistribution of polymeric nanoparticles in the eye and the role of surface properties, size, suspension media, and administration route of nanoparticles on their distribution profile. Thus, this review seeks to analyze the current literature on the biodistribution of polymeric nanoparticles in the eye to elucidate the best performing nanoparticle designs and administration strategies to address specific ocular diseases.

**Fig. 1:**
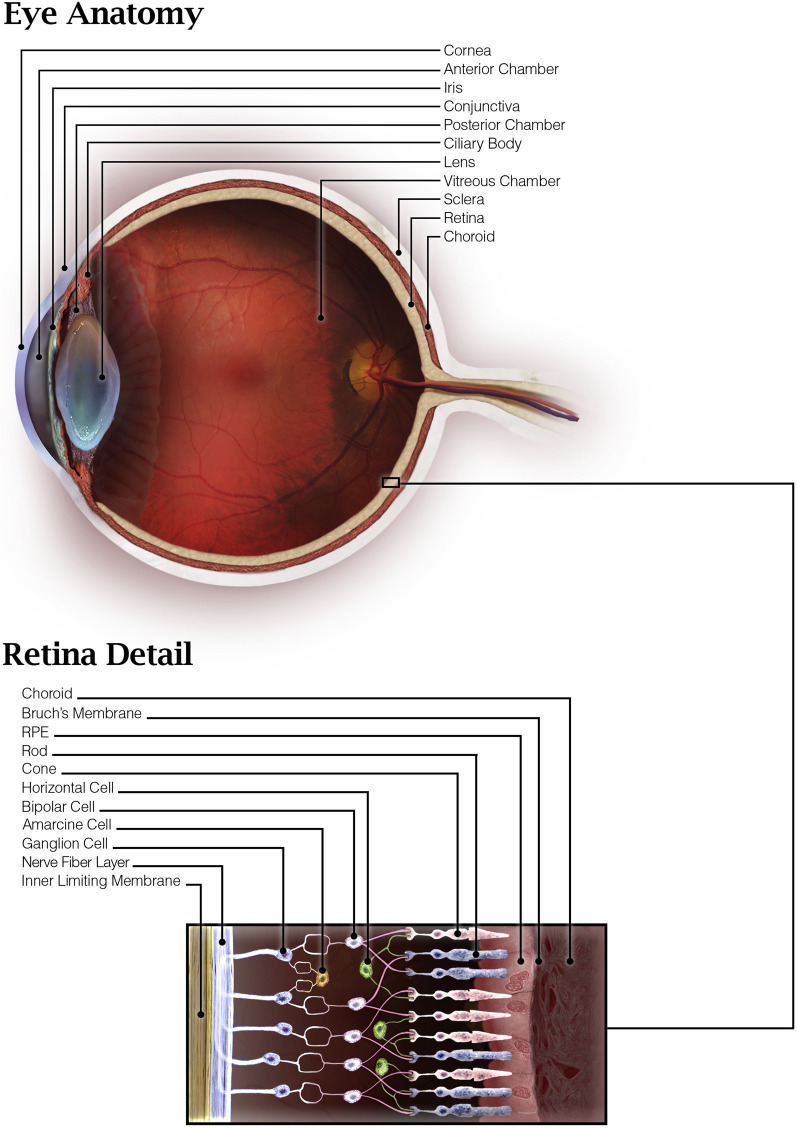
Anatomy of the eye and administration routes

A cross-sectional diagram of the human eye (Fig. 1) reveals the major eye tissues addressed in this review and application routes of nanoparticles intended ocular drug or nucleic acid delivery. While transport of nanoparticles through much of the eye occurs via passive diffusion, there are several barriers that prevent passage unless nanoparticles can utilize transcellular transport due to the presence of tight junctions. The cornea and sclera form a tough barrier to substances outside the eye, the tear film dilutes and eliminates most topically applied solutions, blood barriers in the vasculature prevent certain substances from entering the eye via systemic circulation, and substances in the eye face constant elimination by draining into the systemic circulation. The trafficking pathways taken by polymeric nanoparticles in the eye are influenced by many factors including polymer composition, size, charge, solubility, surface ligands, and administration route. Because of this, the nanoparticle design and administration route must be carefully considered when designing an ocular nanoparticle delivery system based on the intended target.

This review is organized into sections that address major tissues of the eye individually. Each section discusses relevant anatomy and physiology of the tissue, pathways taken by nanoparticles to reach the tissue, studies that have investigated the biodistribution of nanoparticles in the tissue, and clinical application of nanoparticle delivery systems for the tissue. Specific nanoparticle formulations are outlined in Table 1 and clinical applications of polymeric nanoparticle drug delivery systems in the eye are outlined in Table 2. The review ends with future direction and conclusions.

**Table 1:** Polymeric nanoparticle distribution within the eye

Significant areas of distribution	Administration route	Polymer	Coating	Size (nm), Zeta Potential (mV)	Additional features	Suggested clinical use	Ref
Cornea	Topical	PCL	PVA	250, − 20*	Benzalkonium chloride	Controlled release of antibiotic drugs to treat corneal infection	[14]
	Subconjunctival	pRNA	None	10	PLGA-PEG-PLGA thermosensitive hydrogel	Delivery of anti-VEGF aptamer to reduce corneal neovascularization and graft rejection	[18]
Iris, Conjunctiva	Topical	PCL	PF68	165, − 20*	Benzalkonium chloride	Delivery of anti-inflammatory medications to treat iritis Controlled release of NSAIDs, antibiotics, or steroidal drugs to treat conjunctivitis	[14]
Ciliary body	Suprachoroidal injection	Polystyrene^†^	None	20–10,000	Carboxylmethylcellulose (700 kDa) and methylcellulose (90 kDa) solution	Delivery of β-blockers or carbonic anhydrase inhibitors for the treatment of glaucoma	[30]
Lens	Topical	PLGA	Gelatin	170, − 12*	None	Delivery of antioxidants for cataract prevention or chaperone proteins to reverse congenital cataracts and stabilize misfolded crystallin proteins Delivery of anti-VEGF for treatment of CNV	[26]
Choroid, Retina	Topical	PCL	PF68	165, − 20*	None	Delivery of anti-VEGF or corticosteroids for treatment of CNV or diabetic retinopathy Delivery of antioxidants to slow progression of dry AMD	[14]
	Topical	PLGA	Chitosan	170, 35*	None	Delivery of antioxidants to slow progression of dry AMD Delivery of anti-VEGF and/or corticosteroids to treat diabetic retinopathy or CNV	[26]
	Suprachoroidal injection	Polystyrene^†^	None	20–10,000	Hyaluronic acid solution	Delivery of anti-VEGF or corticosteroids for treatment of CNV or diabetic retinopathy Delivery of antioxidants to slow progression of dry AMD	[30]
Retina	Subconjunctival injection	pRNA	None	10	PLGA-PEG-PLGA thermosensitive hydrogel	Delivery of anti-VEGF aptamer to treat diabetic retinopathy	[18]
	Intravitreal injection	PLGA	PVA	227 ± 15, − 2 ± 1	None	Delivery of anti-VEGF and/or corticosteroids to treat diabetic retinopathy	[67]
	Intravitreal injection	Glycosylated chitosan	None	229.1 ± 8.7, 16.4 3.2	None	Delivery of neuroprotective agents to ganglion cells to treat glaucoma	[63]
	Intravitreal injection	CK/PEG-DNA polyplex	None	60 ± 6, − 1 ± 4	None	Gene delivery to the retina to treat inherited retinal degeneration VEGF silencing to treat exudative AMD	[67]
Inner Retina	Intravitreal injection	Hyaluronic acid	None	213.4 ± 10.3, − 26.2 ± 4.0	None	Delivery of anti-VEGF, kinase inhibitors, or gene delivery to silence VEGF for treating diabetic retinopathy	[63]
	Intravitreal injection	Human serum albumin	None	326.3 ± 9.7, − 20.9 ± 2.0	None	Delivery of anti-VEGF, kinase inhibitors, or gene delivery to silence VEGF for treating diabetic retinopathy	[63]

*Data extracted from bar graph. ^†^Polymer used as a proof of concept and is not necessarily intended for drug delivery

**Table 2:** Clinical applications of polymeric nanoparticles in the eye

Disease	Animal model	Polymer, Drug	Size (nm), Zeta potential (mV)	Route	Drug efficacy	Clearance	Toxicity	Ref
Corneal allograft rejection	Rat	PLGA, Dexamethasone sodium phosphate	200 ± 8, − 8 ± 1.4	Subconjunctival injection	Prevented corneal edema and opacification. Significantly reduced neovascularization	65% retained in conjunctiva after 2 d and 5% after 7 d	Mild inflammation at injection site. No corneal inflammation	[23]
	Mouse	PLGA-RGD peptide, Flt23k plasmid	270.2	Subconjunctival injection	Significantly reduced neovascularization and increased graft survival by 20% alone. Combined with triamcinolone, graft survival was increased to 91.6%	NA	NA	[24]
Glaucoma	Rabbit	Hyaluronic acid-modified chitosan, dorzolamide hydrochloride and timolol maleate	319.5 ± 4, 33.3 ± 6.1	Topical	Significantly reduced IOP more than marketed free drug formulation with longer sustained effect	NA	No ocular irritation detected up to 24 h	[33]
Uveitis	Rabbit	Poly beta-amino ester, triamcinolone acetonide	178 ± 6, 5.3 ± 1.7	Topical	Topically applied nanoparticles reduced inflammation to the same degree as subconjunctivally injected free drug	NA	NA	[35]
Autoimmune Uveitis	Rat	PLGA, zinc, Dexamethasone sodium phosphate	210 ± 15, − 9 ± 2	Subconjunctival injection	Reduced clinical disease score, inflammatory cytokine expression, and microglia activation, and improved ERG response compared to free drug	Dexamethasone sodium phosphate, detectable for 21 d post injection	No changes in retinal function or ocular histology due to nanoparticle injection	[36]
Autoimmune Uveoretinitis	Rat	PEG-PLGA, betamethasone	120	Intravenous injection	Reduced inflammatory symptoms up to 14 d	NA	NA	[34]
Selenite Cataract	Rat	PLGA or Zein, lutein	222.9 ± 1.2, − 32.4 ± 3.9	Topical and Oral	Topically applied PLGA and zein nanoparticles loaded with lutein significantly decreased cataract score compared to control, free lutein, and orally administered lutein or lutein loaded nanoparticles	NA	NA	[45]
Posterior lens opacification	Rabbit	Chitosan, 5-fluorouracil	< 400 nm (most < 100 nm)	Loaded into intraocular lens prior to transplantation	nanoparticles significantly decreased proliferation of lens epithelial cells, increased apoptosis, and reduced necrosis compared to free drug loaded lens implants	Burst release for 10 h, followed by sustained released for 100 h	Significantly reduced inflammation and immune cell infiltration compared to free drug loaded lens implants	[46]
Brucellosis	46	Mannosylated-poly(anhydride), hot saline antigen complex	306 ± 11, − 34.6 ± 1.3	Subconjunctival injection	nanoparticle group had twofold higher fecal IgA excretion and significant reduction in spleen CFU compared to standard Rev1 vaccine	NA	No toxicity observed in cornea or iris but hyperemic blood vessels and redness occurred in some eyes	[51]
Choroidal neovascularization	Rat	PLA/PLA-PEO, C16Y integrin antagonist peptide	302.5 ± 85.1, − 38.26 ± 1 .42	Intravitreal injection	Prolonged anti-angiogenic effect longer than free drug (at least 12 d)	NA	No acute inflammatory response	[56]
	Mouse	PLGA microparticles, Serpin-derived peptide	6000	Intravitreal injection	Prolonged anti-angiogenic activity longer than free drug (up to 14 weeks)	NA	NA	[57]
	Rat	PLGA, shRNA targeting (HIF-1α)	303.7 ± 3 8.5	Intravitreal injection	Significantly decreased choidal leakage and thickness of lesions	NA	No changes in histology or retinal function	[58]
Diabetic retinopathy	Mouse	CK_30_PEG_10K_, miR200b	NA	Intravitreal injection	Decreased expression of VEGR-2 and suppressed angiogenesis for 3 months post injection	NA	NA	[59]
Retinal detachment and excitotoxicity	Rat	Poly(γ-glutamic acid)-*L*-phenylalanine, dexamethasone	180 ± 45, − 25	Intravitreal injection	nanoparticles suppressed TNFα and MCP-1 cytokines in cultured macrophages and microglia, reduced microglia activation and death of retinal ganglion cells in excitotoxic animal models, and decreased apoptosis of photoreceptors in retinal detachment animal models	NA	NA	[76]
Acute retinal photo-injury	Rat	PLGA, connexin43 mimetic peptide	NA	Intravitreal injection	Significantly improved ERG response compared to control and decreased immune cell infiltration, reduced astrocyte and Müller cell activation, and preserved choroidal thickness compared to free drug and control	NA	NA	[77]
Optic nerve crush	Rat	HSA, brimonidine	152.86 ± 51.1, − 29. ± 7.5	Intravitreal injection	Significantly reduced A-β deposition in retinal ganglion cells and increased retinal ganglion cell survival	NA	NA	[78]

### Cornea

#### Biodistribution

The cornea is the transparent tissue comprising the anterior surface of the eye and, along with the sclera, forms the outer structure of the eye ball (Fig. 1). Its thickness ranges from 551 to 565 μm in the center and 612–640 μm in the periphery, and it is composed of 5 layers: epithelium, Bowman’s layer, stroma, Descemet membrane, and the endothelium [8]. Pharmacokinetic studies have shown the epithelium and the stroma to be the rate limiting layers for transcorneal permeation of drugs, with the tight junctions of the epithelium forming a barrier to hydrophilic molecules and the stroma, a mostly acellular layer composed primarily of water and collagen fibers, forming a barrier to lipophilic molecules [9]. Under normal physiological conditions, the cornea is avascular; deriving most of its nutrients and hydration from the aqueous humor, tear film, and small blood vessels present at the limbus [8]. While this eliminates systemic blood vessels as a concern for the loss of drugs administered to the cornea, the tear film itself is responsible for a significant loss of topically applied drugs due to tear turnover and adsorption of tear proteins [9].

**Fig. 2:**
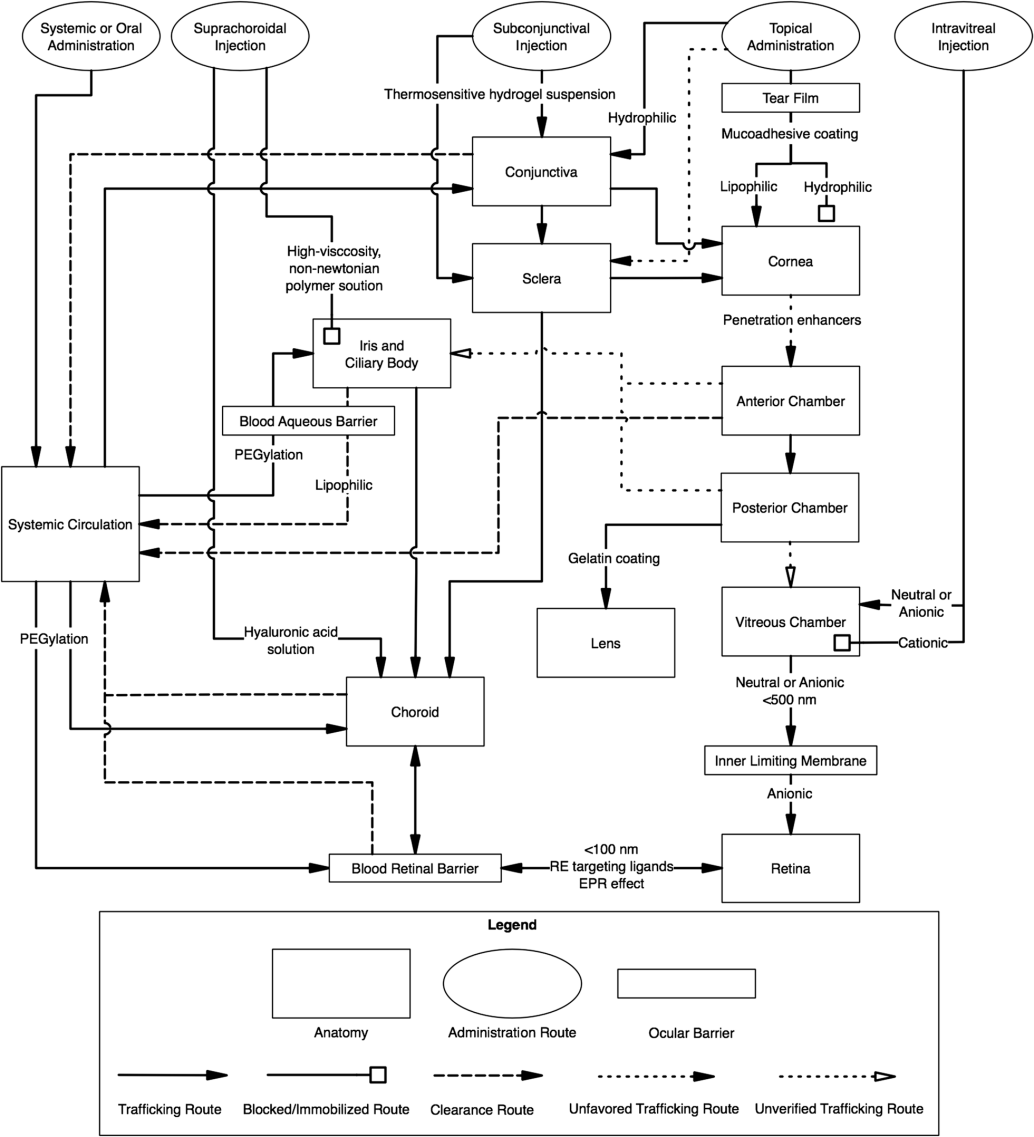
Nanoparticle trafficking routes in the eye

The cornea contains barriers to both hydrophilic and hydrophobic molecules, and the pore size of the corneal epithelium is approximately 2 nm with a relatively low pore density compared to the conjunctiva, requiring most nanoparticles to permeate through via the trancellular pathway [9–11]. Additionally, the surface area of the cornea is about 17 times smaller than the conjunctiva, making it generally less available to permeation of applied substances than the conjunctiva (Fig. 2) [12]. Nevertheless, some topically administered nanoparticles, including poly(lactic-co-glycolic acid) (PLGA) and poly(caprolactone) (PCL) nanoparticles, are able to penetrate the cornea and enhance topical drug delivery [13]. Both PLGA and PCL are formed by ester linkages between monomers and carry end carboxylic acid groups unless modified. As such, nanoparticles formed with these polymers typically carry a negative surface charge and will degrade in water due to hydrolysis of the ester bonds. The use of mucoadhesive nanoparticle coatings such as Pluronic-68 (PF68) and chitosan, a positively charged polysaccharide derived from chitin, can enhance corneal residence time and epithelial surface contact of topically applied nanoparticles by interacting with mucins secreted in the tear film, increasing the likelihood of endocytosis and trancellular transport [11, 14]. Additionally, penetration enhancers can act on cell membranes and tight junctions to temporarily improve corneal permeability, especially for hydrophilic particles with limited potential for transcellular permeation [11, 15]. PCL nanoparticles, when administered alongside benzalkonium chloride, an ocular penetration enhancer showed higher distribution to the cornea than other PCL nanoparticle formulations with and without mucoadhesive coatings and various penetration enhancers (Table 1) [14]. On the other hand, PCL nanoparticles coated with PF68 showed significantly higher distribution to the iris, suggesting greater transcorneal diffusion (Fig. 2), with penetration enhancers further increasing this effect (Table 1) [14]. While penetration enhancers may improve the transcorneal diffusion of topically applied nanoparticles, adverse effects have been reported in the cornea and other eye tissues, especially with repeated doses, and further testing is necessary to optimize both safety and treatment efficacy [15]. An alternative to topical application for delivery to the cornea is subconjunctival injection. This method prevents precorneal loss of drugs to the tear film; however, rapid clearance via systemic and lymphatic circulation still remains an issue [16, 17]. Guo et al. found that, when suspended in a thermosensitive hydrogel composed of PLGA and poly(ethylene glycol) (PEG) copolymer (PLGA-PEG-PLGA), the clearance of pRNA (packaging RNA) nanoparticles injected into the subconjunctival space is significantly slowed and the concentration of nanoparticles in the cornea is significantly higher after 36 h than nanoparticles that were suspended in PBS [18].

#### Clinical applications

Because it forms the outer, anterior surface of the eye, traumatic injury to the cornea is a common pathology and can result from physical trauma or from chemical and thermal burns. While more superficial injuries to the cornea can heal without complication due to the regenerative ability of the epithelial layer, injuries that penetrate deeper into the cornea cannot easily heal and result in scarring which can impair vision. Long-term use of contact lenses can also result in corneal complications including scarring. Serious corneal injuries and severe scarring are treated via transplantation of a corneal allograft. Unfortunately, graft rejection occasionally occurs, especially in high risk populations, which often results in an additional serious corneal pathology: corneal neovascularization [19, 20] as immune cells infiltrate the injury site and begin releasing angiogenic factors such as vascular endothelial growth factor (VEGF) [21, 22]. Corneal neovascularization disrupts the transparency of the cornea, resulting in vision loss, and often requires an additional graft surgery if it occurs as a complication of graft rejection [19].

The cornea, being an outermost tissue of the eye, can be easily reached by drugs with both topical and subconjunctival administration. Nevertheless, nanoparticles can offer the advantage of controlled drug release for prolonged effects in the cornea relative to other formulations. Indeed, it has been shown that PLGA nanoparticles loaded with dexamethasone sodium phosphate (DSP), exhibiting sustained drug release for approximately 16 d, were able to sustain ocular drug levels for 7 d after a single dose when administered subconjunctivally, effectively preventing corneal allograft rejection in rats when administered weekly for a 9-week period, while animals treated with free DSP experienced graft rejection, cornea neovascularization, edema, and opacity (Table 2) [23]. In some cases, the multifunctionality of drug loaded nanoparticles sets them apart from free drug treatments when the therapeutic effects are comparable or lower. Cho et al. tested the ability of PLGA nanoparticles with conjugated arginine-glycine-aspartic acid (RGD) peptides to deliver Flt23k genes (an anti-VEGF intraceptor) to the cornea for enhanced graft survival and reduced neovascularization in rats (Table 2) [24]. Animals treated with the gene loaded nanoparticles experienced significantly higher graft survival rates than the controls, though not as high as animals treated with triamcinolone acetonide, a potent corticosteroid [24]. However, the nanoparticles greatly reduced neovascularization of the graft while triamcinolone increased it, and a combination of the nanoparticles and triamcinolone increased graft survival rate up to 91.6% and reduced neovascularization of the graft compared to only triamcinolone (Table 2) [24].

### Iris and ciliary body

#### Biodistribution

The iris is a thin pigmented structure of the eye that regulates the amount of light entering the eye by constricting and dilating the pupil (Fig. 1). It is composed of a pigmented stromal layer and underlying pigmented epithelial cells. The ciliary body includes ciliary muscles, which are responsible for visual accommodation by changing the shape of the lens via zonule fibers, and ciliary epithelial cells which produce aqueous humor, the transparent protein containing fluid that nourishes the cornea and lens [25]. Together, the iris, ciliary body, and choroid form the uvea.

Studies assessing the ocular biodistribution of topically applied nanoparticles have shown a relatively low distribution to the iris and ciliary body [14, 26]. This is likely due, in part, to the challenges of transcorneal diffusion of topically administered nanoparticles. Nanoparticles that are able to penetrate the cornea and enter the anterior chamber have to diffuse against the flow of aqueous humor and face elimination via Schlemm’s Canal (Fig. 2) [27, 28]. Nanoparticles that successfully reach the anterior uvea once again face elimination if they cross the blood-aqueous barrier into the uveal circulation, especially for lipophilic particles (Fig. 2) [28]. Nevertheless, topically administered PCL nanoparticles coated with PF68 and administered with benzalkonium chloride distributed significantly more to the iris compared to other tested formulations (Table 1), albeit much less than other tissues of the eye [14]. Due to the relatively low distribution of topically administered nanoparticles to the iris/ciliary body, high doses would be required to achieve therapeutic effects which increases the risk of adverse effects in other tissues [29]. Therefore, topically applied nanoparticles may not be a good candidate for delivery to the iris and ciliary body. Injection into the suprachoroidal space is likely a more effective approach for nanoparticle-based drug delivery to the ciliary body (Fig. 2) [30]. Kim et al. found that, when combined with non-Newtonian polymer solutions with high viscosity at low shear forces (carboxylmethylcellulose and methylcellulose), polystyrene nanoparticles and microparticles injected into the suprachoroidal space are immobilized directly adjacent to the ciliary body, providing a potentially highly effective method for targeted drug delivery to the ciliary body (Table 1) [30].

#### Clinical applications

Aqueous humor is secreted by the ciliary epithelial cells into the posterior chamber, flows anteriorly into the anterior chamber, and flows outwards through the trabecular meshwork into Schlemm’s canal where it drains into systemic circulation (Fig. 1) [27]. The constant secretion, flow, and drainage of aqueous humor controls the intraocular pressure (IOP) of the eye. Changes in the eye that block the outflow of aqueous humor cause an increase in IOP which can result in the death of retinal ganglion cells and irreversible blindness if sustained [31]. This can result from changes in the trabecular meshwork, slowing drainage of the aqueous humor, or contact between the iris and lens, blocking the humor from flowing into the anterior chamber [31]. While the ciliary epithelial cells themselves are not responsible for glaucoma, they are a main target for its treatment, using β-blockers, such as timolol maleate, or carbonic anhydrase inhibitors, such as dorzolamide hydrochloride, to reduce aqueous humor secretion and subsequently the IOP [31]. Uveitis is another common pathology affecting the iris and ciliary body, which, together with the choroid, form the uvea. Uveitis is characterized by inflammation in the uvea and can be caused by infection, trauma, toxic exposure in the eye, or autoimmunity [32]. Uveitis can result in pain, redness, sensitivity to light, and blurred vision, and is typically treated with corticosteroids to reduce inflammation to control the inflammation and immunosuppressive drugs if caused by an autoimmune response.

Topically administered dorzolamide hydrochloride and timolol maleate have been shown to significantly reduce IOP in rabbits more and with a longer sustained effect when loaded in hyaluronic acid-modified chitosan nanoparticles compared to a marketed free drug formulation (Table 2) [33]. Polymeric nanoparticles loaded with various corticosteroids and administered through multiple routes including intravenous, topical, and subconjunctival, have been demonstrated to effectively reduce inflammation in animal models of uveitis [34–36]. When administered intravenously, nanoparticles formed from a co-polymer of poly(lactic acid) (PLA) and PEG, loaded with betamethasone phosphate, could effective distribute to the eyes of rats with experimental autoimmune uveoretinitis and reduce inflammatory symptoms for up to 14 d (Table 2) [34]. The concentration of betamethasone phosphate in the eyes of animals who received intravenous injection of PLA-PEG nanoparticles was sixfold higher than animals that received PLA nanoparticles loaded with betamethasone phosphate [34]. This phenomenon is certainly due, in part, to the “stealth” properties of PEG, a hydrophilic and biologically inert polyether, which reduces protein adsorption in the blood and improves distribution of the nanoparticles to tissues when administered systemically [34, 37].

### Lens

#### Biodistribution

The lens is a transparent biconvex structure located behind the iris in the posterior chamber, and it functions to transmit and focus light onto the retina (Fig. 1). The lens has no blood supply after fetal development and derives its nutrients from the aqueous humor. It is composed of 4 structures: the lens capsule, epithelium, cortex, and nucleus. The lens capsule is the outermost tissue layer of the lens, forming an acellular basement membrane, and attaches to the ciliary body via zonular fibers. There is a single layer of epithelial cells inside the capsule lining the anterior surface of the lens, forming a barrier to hydrophilic molecules. These cells are responsible for maintaining homeostasis of the lens by regulating water and ion transport through the lens. Additionally, the epithelial cells serve as progenitors for lens fibers, organelle lacking cells that contain crystallin proteins and comprise most of the volume of the lens. The cortex and nucleus are composed entirely of lens fibers, whose tightly compact arrangement limit drug diffusion in the lens. These cells contain organized, water-soluble crystallin proteins and lack organelles to allow for the transparency of the lens. Disruptions in the structure of these fibers and proteins can lead to a loss of transparency [38].

Topical drug delivery to the lens faces the same challenges as the iris and ciliary body because it requires corneal penetration and diffusion through the aqueous humor (Fig. 2). PCL nanoparticles formulations with various mucoadhesive coating and penetration enhancers did not effectively reach the lens 1 h after administration [14]. However, a follow-up study showed that gelatin coated PLGA nanoparticles distributed well to the lens and persisted for up to 4 h while PLGA nanoparticles coated with chitosan or PF68 did not effectively distribute to the lens [26]. The authors posit that this could be a result of RGD ligands in the gelatin binding to RGD-binding β_1_ integrins on lens epithelial cells [26]. An additional barrier of drug delivery to the lens is the lens capsule. Schachar et al. found that cadmium tellurium nanoparticles were unable to diffuse through the porcine lens capsule despite being smaller 10 nm, while dextran molecules have been shown to readily diffuse through the lens capsule [39, 40]. The authors suggest this could be a result of carboxylic acid groups present of the surface of the nanoparticles, creating a negative surface charge [39]. If this is the case, polymeric nanoparticles with a negative surface charge would be expected to have trouble reaching the inner lens, though this does not appear to be the case in studies that detect fluorescent PLGA nanoparticles in the lens of rats following topical administration [26, 41].

#### Clinical applications

The most common pathology of the lens is cataract formation, which is characterized by a reduction in the optical clarity of the lens. There are several causes of cataracts, including aging, heredity, trauma, and diabetes. As the lens ages compression and hardening occurs, especially in the nucleus, inhibiting the transport of water, ions, and antioxidants through the lens. Because the lens is frequently under photo-oxidative pressure, this makes crystallin proteins susceptible to post-translational modifications including oxidation, which causes the proteins to precipitate and aggregate, resulting in light scatter and loss of transparency [38, 42]. In the case of diabetic cataracts, increased levels of reducing sugars present in the lens cause glycation of the crystallin proteins which also leads to a cataract [43]. Additionally, sorbitol accumulation in the lens of diabetic patients has a hyperosmotic effect, causing the lens to swell. This osmotic stress can result in the death of lens epithelial cells and formation of reactive oxygen species (ROS), causing oxidative stress and subsequently cataract formation [43]. Unlike diabetic and senile cataracts, congenital cataracts are inherited, develop much earlier in life, and can be caused by several mutated proteins including αA-crystallin protein which helps maintain the crystalline structure of the lens through chaperone-like activity [44].

Due to the nature of cataract formation, involving irreversible changes to crystallin protein structure, they are incurable without surgical replacement of the cloudy lens. Therefore, nanoparticle-based therapies can be directed towards cataract prevention or post-operative care. Bodoki et al. tested the ability of PLGA and zein nanoparticles loaded with lutein, a carotenoid antioxidant in macular pigment, to attenuate selenite-induced cataracts in rats when administered orally or topically, and found that both topically applied nanoparticles, PLGA and zein, reduced cataract progression at several tested concentrations, whereas topically applied free lutein and orally administered formulations of free lutein and nanoparticles had no significant therapeutic effect [45]. The most common complication of lens replacement surgery is posterior capsule opacification (PCO), which occurs when lens epithelial cells along the anterior lens capsule proliferate, undergo epithelial to mesenchymal transition (EMT), migrate posteriorly, and deposit collagens. Huang et al. set out to devise a way to prevent PCO by incubating the intraocular lens in either 5-fluorouracil, an anti-cancer drug, or nanoparticles loaded with loaded free 5-fluorouracil [46] prior to implantation in rabbits. Both 5-fluorouracil loaded nanoparticles and free 5-fluorouracil prevented cloudiness of the implant to a similar degree, but free 5-fluorouracil induced intraocular inflammation while the nanoparticles did not [46].

### Conjunctiva and sclera

#### Biodistribution

The conjunctiva is a mucous membrane extending outward from the corneal limbus, lining the anterior exposed sclera and interior aspect of the eyelids (Fig. 1). Drug diffusion through the conjunctiva is primarily limited by the epithelium, much like the cornea, as it is composed of nonkeratinizing squamous cells, cuboidal basal cells, and goblet cells [47]. The conjunctiva runs 3–5 cell layers thick, with tight junctions on the apical surface [47]. The sclera is the fibrous, opaque tissue forming the outer layer of the eye and is continuous with the cornea (Fig. 1). It is composed primarily of collagenous and elastic fibers with an inner endothelial lining, like the cornea, and has a varying thickness throughout the eye [48].

Most topically applied nanoparticles are absorbed into the conjunctiva due to its large surface area and greater permeability than cornea. The tight junctions between epithelial cells of the conjunctiva are leakier than the cornea [12]. Rabbit conjunctiva has twice the pore size than the cornea with a 16 times higher pore density, allowing for 15–25 times higher permeability to PEG-oligomers than the cornea, while the sclera was demonstrated to be 10 times more permeable to PEG-oligomers than the cornea and half as permeable as the conjunctiva [10]. A study assessing the biodistribution of topically administered PCL nanoparticles with and without mucoadhesive coating and penetration enhancers showed that all formulations distribute well to the conjunctiva compared to other tissues, and PCL nanoparticles coated with PF68 and administered with benzalkonium chloride distribute significantly greater to the conjunctiva compared to the other formulations (Table 1) [14].

The periocular, or transscleral, permeation of topically applied, sirolimus-loaded Cholesterol-PEG nanoparticles (12–16 nm) were compared to sirolimus dissolved in dimethyl sulfoxide (DMSO) [49]. Permeation of sirolimus dissolved in DMSO was 14 times higher than nanoparticles; however, nanoparticles did not induce any histological changes in the tissue while the DMSO solution increased spacing of the collagen fibrils. When applied topically, doxorubicin, an anticancer drug, was able to permeate across the sclera much faster in free solution than when loaded in PLGA nanoparticles of approximately 265 nm [50]. While topically applied, polymeric nanoparticles may not be able to permeate through the sclera as quickly as free drugs in solution due to their size, sub-conjunctival injection can improve their periocular permeation by keeping the nanoparticles in contact with the sclera longer. Due to the vascular nature of the conjunctiva, strategies should be employed to reduce the systemic clearance of nanoparticles injected into the subconjunctival space. The use of thermosensitive hydrogels as a suspension media for nanoparticles can reduce their systemic clearance when injected into the subconjunctival space due to the increased viscosity of the suspension as it heats up in contact with the tissue, effectively improving periocular delivery and bioavailability in the inner eye [18].

#### Clinical applications

While topically applied nanoparticles distribute well to the conjunctiva compared to other tissues of the eye, there are few pathologies of the conjunctiva and sclera that call for advanced delivery systems such as polymeric nanoparticles. Nevertheless, Martins et al. tested an interesting application of polymeric nanoparticles in the conjunctiva: a vaccine delivery system [51]. Martins et al. designed a vaccine against *Brucella ovis* using mannosylated poly(anhydride) nanoparticles loaded with hot saline antigen complex isolated from *B. ovis* bacteria [51]. Conjunctival installation of the vaccine was shown to increase IgA excretion and reduce spleen colony forming units (CFU) significantly more than control animals and animals who received intraperitoneal injection of Rev1, the golden standard vaccine for *B. ovis* (Table 2) [51].

### Choroid

#### Biodistribution

The choroid is a layer of vasculature lying between the retina and sclera which provides blood supply to the outer retina layers (Fig. 1). Nanoparticles that enter the choroidal vessels travel posteriorly to the vortex veins to be cleared from the eye. The innermost layer of the choroid, Bruch’s membrane, contains several layers of collagenous and elastic fibers, and forms the basement membrane for retinal pigment epithelium (RPE). Bruch’s membrane thickens as an individual ages, slowing the diffusion of metabolites and waste between the choroid and RPE, which may lead to the development of drusen, a hallmark sign of age-related macular degeneration (AMD) [52]. Choroidal neovascularization (CNV), often referred to as “wet” AMD, is strongly associated with drusen deposits in patients with AMD [53]. Though not all patients with AMD will develop CNV, retinal degeneration and the loss of vision occurs more quickly compared to patients with “dry” AMD. Oxidative stress and inflammation via compliment activation stimulate VEGF-A secretion by RPE cells, which triggers angiogenesis of the choroid [54].

Topically administered nanoparticles can reach the choroid by either penetrating the conjunctiva and entering the sclera or by directly penetrating the sclera. While the relatively leaky nature and large surface area of the conjunctiva makes this easily achievable, rapid clearance from the choroid via systemic circulation is a larger concern for drug delivery. Topically administered PCL nanoparticles coated with PF68 distributed significantly more to the choroid in 1 h than PCL nanoparticles coated in gelatin or chitosan [14]. Interestingly, in a follow-up study, gelatin coated PLGA nanoparticles distributed more effectively to the choroid than gelatin coated PCL nanoparticles, and chitosan coated PLGA nanoparticles showed the greatest distribution to the choroid, followed by gelatin, with PF68-coated PLGA nanoparticles showing the least distribution to the choroid (Table 1) [26]. All formulations tested in the follow-up study appeared to persist for at least 4 h [26]. Overall, topically administered nanoparticles distribute relatively well to the choroid compared to other inner eye structures, likely due to the multiple entry points (Fig. 2). Nevertheless, injection of nanoparticles into the suprachoroidal space is a more direct method for delivering nanoparticles to the choroid (Fig. 2) [30]. The inclusion of a hyaluronic-acid solution enhances the spreading of particles throughout the suprachoroidal space and slows clearance, thereby enhancing delivery of nanoparticles to the choroid (Table 1) [30].

#### Clinical applications

Choroidal neovascularization (CNV), also known as wet or exudative AMD, is less common than the dry form of AMD but progresses much more rapidly. Intravitreal injection of angiogenesis inhibitors is an effective way to manage CNV, but long term use of frequent intravitreal injections can result in adverse effects such as retinal detachment [55]. Therefore, a goal of polymeric nanoparticle systems for treating CNV is to prolong the drug effect via controlled release, effectively reducing the frequency of injections and adverse effects. Indeed, PLA/PLA-Poly(ethylene oxide) nanoparticles loaded with an integrin antagonist peptide showed similar anti-angiogenic activity to free drug 9 d post intravitreal injection in rats with CNV, but the nanoparticles showed significantly greater anti-angiogenic activity compared to the free drug at 12 d post injection, indicating a sustained drug effect due to the release from nanoparticles which was sustained for over 6 weeks in vitro (Table 2) [56]. Unlike smaller nanoparticles, intravitreally injected microparticles are likely immobilized in the vitreous humor due to their size. Nevertheless, it has been shown that PLGA microparticles loaded with serpin-derived peptide, and angiogenesis inhibitor, were able to sustain therapeutic effects up to 14 weeks after a single dose while the therapeutic effect of free peptide only lasted for 4 weeks, likely due to the sustained release of serpin-derived peptide from microparticles, which was sustained for over 200 d in situ, in the vitreous body followed by released serpin-derived peptide diffusing to the choroid (Table 2) [57].

In addition to anti-angiogenic drug delivery, polymeric nanoparticles can be used for gene delivery to the choroid for CNV treatment. Zhang et al. tested PLGA nanoparticles for delivery of a plasmid DNA for transfection of short hairpin RNA (shRNA) against hypoxia inducible factor -1α(HIF-1α), which has implications in VEGF overexpression leading to CNV. GFP expression, indicating successful transfection, was detectable in the RPE for up 28 d after intravitreal injection, compared to only 7 d when the naked plasmid was injected [58]. As a result, rats with CNV had significantly less leakage in CNV membranes and reduced thickness of CNV lesions 14 d after treatment with the plasmid loaded nanoparticles compared to naked plasmid [58] (Table 2). CK_30_PEG_10K_ compacted DNA nanoparticles have also been tested for suppressing angiogenesis in mice with diabetic retinopathy via delivery of miR200b, a potent inhibitor of VEGFR-2 expression, finding that mice treated with the nanoparticles showed a remarkable decrease in angiogenesis and VEGFR-2 expression which lasted for up to 3 months after a single intravitreal injection [59] (Table 2). CK_30_PEG_10k_ is part of a family of polylysine-based nanoparticles used to compact DNA for non-viral gene transfection [60]. In this case, the 30-mer polylysine was conjugated with a 10 kDa PEG via maleimide linkage and used to compact the miR200b-containing plasmid DNA [59].

### Retina

#### Biodistribution

**Fig. 3:**
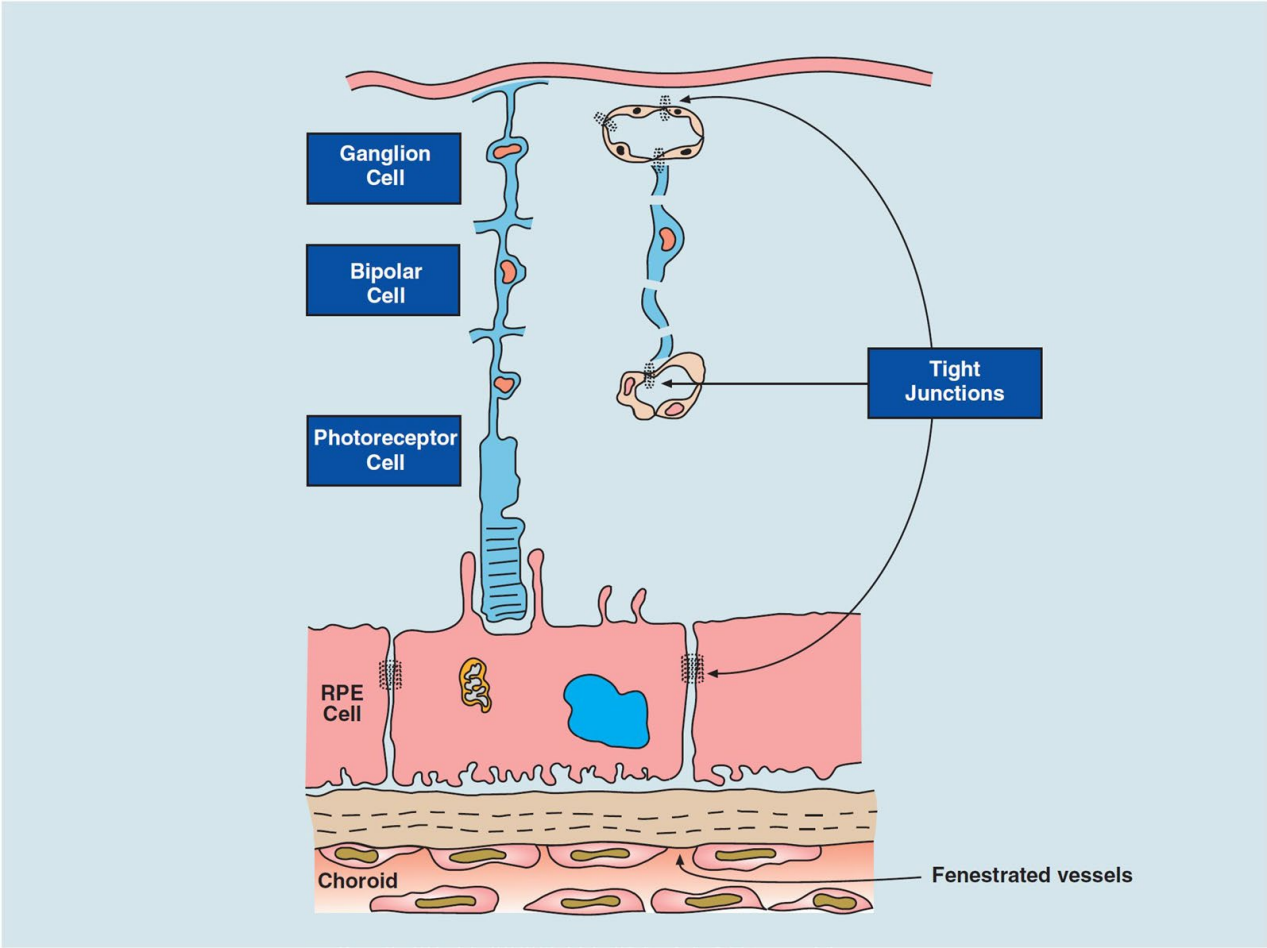
Blood-retinal barrier [65]. Permission to reproduce this figure is granted under the Creative Commons Attribution License. Figure 3 is reprinted from Frontiers in Immunology, Forrester V & Xu H, Good news–bad news: the Yin and Yang of immune privilege in the eye, 2012, under the Creative Commons Attribution License

The retina lines the back of the eye (Fig. 1), and functions by detecting light that enters the eye and converting it to a signal which travels to the brain via the optic nerve. Retinal ganglion cells comprise the innermost cellular layer of the retina while photoreceptors form the outer layer in contact with the RPE [61]. The eye is similar to the brain in that it is sequestered from certain blood components such as antibodies and immune cells [62]. This is in part due to the presence of the blood-retinal barrier (BRB), which is physiologically similar to the blood–brain barrier and, like the brain, can complicate drug delivery strategies. The inner BRB is composed of tight junctions between retinal capillary endothelial cells, while the outer-blood retinal barrier is formed by tight junctions between RPE cells, separating the choroid and Bruch’s membrane from the inner retina (Fig. 3) [62]. The end feet of Müller cells, glial cells similar to astrocytes, form the inner limiting membrane, which creates barrier between the vitreous humor and inner retina [61]. Therefore, drugs and nanoparticles intended to reach the retina must be able to cross the inner limiting membrane or BRB depending on the path taken (Fig. 2). With an average pore size of 10–25 nm, the inner limiting membrane creates a strict physical barrier for most polymeric nanoparticles; however, uptake and transcellular permeation via Müller cells may be an alternative mechanism to reach the inner retina from the vitreous chamber [63, 64].

**Fig. 4:**
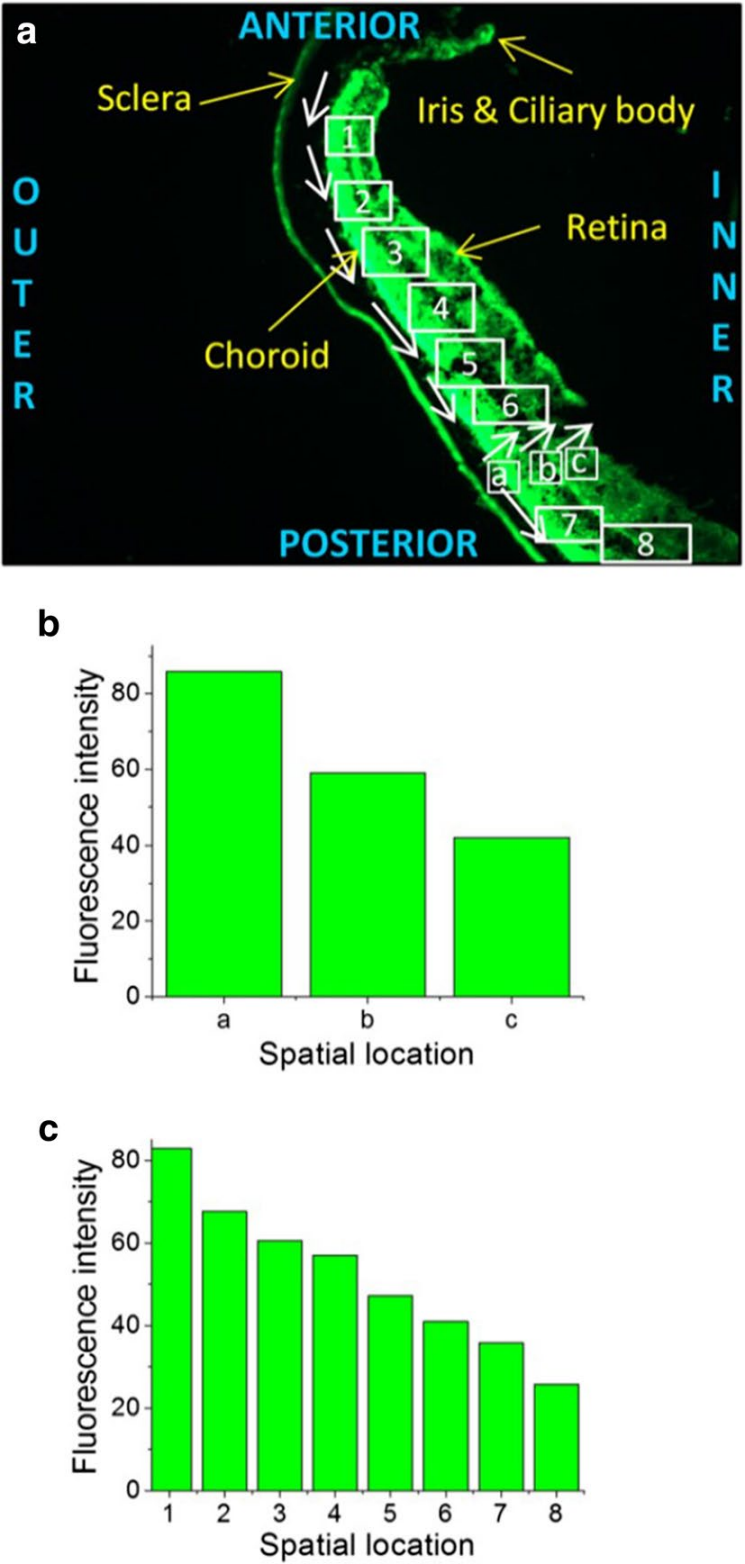
**a** Retinal cross section of mouse eye showing gradients of fluorescence due to movement of nanoparticles, **b** Outer to inner gradient in fluorescence **c** Anterior to posterior gradient in fluorescence [26]. Permission to reproduce this figure has been obtained from Elsevier. Figure 4 is reprinted from Nanomedicine: Nanotechnology, Biology, and *Medicine*, 12/7, Mahaling B & Katti DS, Physicochemical properties of core–shell type nanoparticles govern their spatiotemporal biodistribution in the eye, 2149–60, 2016, with permission from Elsevier

Topically administered nanoparticles can reach the retina via periocular permeation, though nanoparticles would have to penetrate the outer BRB upon diffusion through the sclera and choroid and reaching the RPE. To reach the most posterior sections of the inner eye, such as the macula region, nanoparticles most likely diffuse around the globe of the eye rather than directly through the anterior, posterior, and vitreous chambers, as the later requires corneal penetration and diffusion directly against the flow within the fluid-filled chambers (Fig. 2). Furthermore, nanoparticles with a positive surface charge are unable to effectively diffuse through vitreous humor, yet chitosan-coated PLGA nanoparticles have been shown to diffuse relatively well to the retina when administered topically, suggesting another route [26]. In support of this, a study by Mahaling et al. found two gradients of fluorescence, indicating the presence of nanoparticles, when measuring the spatiotemporal distribution of topically administered polymeric nanoparticles in the eye: from anterior to posterior and from outer to inner eye (Fig. 4) [26]. Unfortunately, many nanoparticles absorbed topically will be eliminated by entering the systemic circulation when crossing the conjunctiva or upon reaching the retina (Fig. 2) [12, 28]. Topically applied PCL nanoparticles coated with PF68 showed the greatest distribution to the retina compared to other PCL nanoparticle formulations, and topically applied PLGA nanoparticles coated with gelatin, PF68 or chitosan show an even greater distribution to the retina compared to PCL nanoparticles, with the greatest concentrations observed in chitosan coated PLGA nanoparticles [14, 26] (Table 1). Subconjunctivally injected nanoparticles likely follow a similar path to reaching the retina as topically administered nanoparticles, and the use of thermosensitive hydrogels to slow clearance can significantly increase the distribution of injected nanoparticles to the retina [18]. While nanoparticles are able to reach the inner retina when administered topically or via subconjunctival injection, higher doses may be required to achieve therapeutic effects in the retina due to the high percentage of nanoparticles that will not reach the retina due to clearance, distribution to other tissues, or lack of penetration into the inner eye [66].

The bioavailability of drug loaded nanoparticles in the retina can be improved using intravitreal injection; however, this administration route is invasive and carries the risk of rare, but serious, complications. PEG-coated polystyrene beads were used to demonstrate that neutrally charged nanoparticles up to 750 nm can readily diffuse through bovine vitreous humor to reach the retina, with greater diffusion coefficients observed in nanoparticles 100–500 nm than 750 nm [67]. Negatively charged beads coated with carboxylic groups can also readily diffuse through vitreous humor, but are more affected by size than neutrally charged nanoparticles as 500 nm did not effectively diffuse [67]. Other negatively charged particles such as PLGA coated in poly(vinyl alcohol) (PVA) (227 nm) and, CK_30_PEG_10K_/DNA polyplex nanoparticles can diffuse to the retina when injected intravitreally (Table 1) [67]. Nanoparticles formed from hyaluronic acid, human serum albumin (HSA), or a complex of the two can reach the retina and penetrate the inner limiting membrane via Müller cells when injected intravitreally (Table 1) [63]. Positively charged polyethyleneimine nanoparticles cannot diffuse through the vitreous humor when injected intravitreally and are therefore not effective for drug delivery to the retina via this administration route [63]. Nanoparticles formed from glycosylated chitosan (200–500 nm) have their positive charge masked by the glycol groups and can reach the retina when injected intravitreally but not penetrate inner limiting membrane (Table 1) [63].

For systemically or orally administered nanoparticles to reach the inner retina, they must be able to penetrate the inner BRB (Figs. 1 and 2). Kim et al. demonstrated that systemically administered gold nanoparticles were able to penetrate the inner BRB at 20 nm but not at 100 nm, suggesting a relatively strict size exclusion for nanoparticles; however, surface ligands targeting the retinal endothelium could promote uptake and transport across the BRB [68, 69]. Pathologies that affect the retinal vasculature integrity, especially CNV, could enhance BRB penetration due to the enhanced permeability and retention (EPR) effect, which is typically associated with tumors (Fig. 2) [69]. Temporarily modulating the BRB to enhance permeability may be another strategy for improving systemic drug delivery to the eye; however, it has yet to be tested with nanoparticles and carries some safety risks [62]. Unfortunately, additional challenges exist for systemically administered nanoparticles, as nanoparticles larger than 200 nm can be subject to scavenging by Kupffer cells in the liver and macrophages in the spleen, and protein adsorption to the surface of the nanoparticle, forming a corona, can mask surface ligands and alter the zeta potential [69–71]. PEG can be used to provide “stealth” properties to nanoparticles, reducing scavenging and corona formation and improving the systemic delivery approach (Fig. 2) [37].

#### Clinical applications

RPE dysfunction and atrophy is a preeminent mechanism in the pathophysiology of AMD, both the dry and wet forms [54]. While the wet form of AMD progresses more rapidly than the dry form, the dry form is much more common [72]. Unfortunately, there is no clear target for drugs in cases of dry AMD, unlike VEGF antagonists in the wet form, and as a result, most studies involving nanoparticles for drug delivery to treat AMD are for the wet form. Nevertheless, oxidative stress is heavily implicated in the pathogenesis of dry AMD, making antioxidant therapy a potential candidate for nanoparticle delivery systems [54, 73]. This approach was addressed by Mo et al. in a study that assessed the potential for HSA nanoparticles to deliver the superoxide dismutase (SOD1) gene to ARPE-19 cells in vitro and mouse retinas in vivo, finding that the transfection efficiency of the nanoparticles was sixfold higher compared to lipofectamine, a commercially available transfection reagent, and SOD1 overexpression was detectable in mouse retinas 2 d after intravitreal injection [74]. Unfortunately, SOD1 expression was not detectable via western blot 7 d after injection of the nanoparticles, and a more advanced gene delivery system may be required to prolong SOD1 overexpression for clinical applications [74]. Nanoparticles composed of polymerized αB-crystallin and elastin-like polypeptides have also been demonstrated to reduce oxidative stress-induced apoptosis in RPE cells though chaperone function [75].

Inflammation also plays a large role in the pathogenesis of AMD as well as other retinal disorders including retinal detachment, eye injury, excitotoxicity, and acute photo-injury, and if not properly controlled can lead to death of photoreceptors and retinal ganglion cells resulting in blindness [76, 77]. Poly(γ-glutamic acid)-*L*-phenylalanine nanoparticles loaded with the corticosteroid, dexamethasone, have been demonstrated to selectively distribute to the retina under pathological conditions when injected intravenously, suppress microglia activation in excitotoxic rat retinas, and reduce apoptosis of retinal ganglion cells and photoreceptors in rats with retinal detachment (Table 2) [76]. Another study investigated the ability of PLGA nanoparticles loaded with connexin43 mimetic peptide (Cx43MP) to reduce inflammation in the retinas of rats with photo-induced retinal damage, finding that intravitreally injected nanoparticles and free Cx43MP both preserved retinal function assessed by electroretinography (ERG) to a similar degree, but the Cx43MP loaded nanoparticles preserved choroidal thickness, decreased immune cell infiltration, and reduced astrocyte and Müller cell activation significantly more than free Cx43MP (Table 2) [77].

## Future directions

While this review outlines some effective approaches for nanoparticle-based drug delivery to the eye, including nanoparticle design and administration route, there are still many gaps in the literature regarding optimal design and trafficking pathways within the eye. Specifically, additional research is warranted to unveil the mechanisms for transport across certain barriers of the eye including the inner-limiting membrane, cornea, BRB, and lens capsule, to determine safe and optimal nanoparticle designs to penetrate these barriers. In addition, rapid clearance remains a challenge for polymeric nanoparticles as they need to release their payload before being eliminated from the eye. While some studies in this review have addressed clearance of nanoparticles from the eye, the times elapsed were, in some cases, not long enough to adequately characterize clearance, and only a few studies included techniques for reducing clearance from the eye [18, 26, 30]. Finally, the clinical relevance of ocular studies using rodent models is highly questionable, especially when quantifying distribution and kinetic properties of nanoparticles in the eye, as there are many sizable differences between the rodent and human eye. Therefore, the most impactful future studies on this topic will come from larger animal models with more physiologically and anatomically similar eyes to our own.

## Conclusion

There are some major challenges to designing, interpreting, and compiling research on the ocular biodistribution of polymeric nanoparticles: (1) There are countless ways to design a nanoparticle-based ocular delivery system, and any change to the size, charge, surface ligands, release profile, suspension media, and administration route may greatly impact its biodistribution and performance in the eye. (2) There are various animal models that have and can be used to test ocular biodistribution. The unique anatomy and size of each species’ eye likely impacts the distribution profile of administered nanoparticles which makes comparing the limited available literature difficult. (3) There are multiple ways to track nanoparticles in eye, and each has advantages and disadvantages. Physically entrapping markers such as fluorophores in nanoparticles can often achieve high levels of brightness, but the release of the fluorophore from the particle may reduce the accuracy and integrity of the data as well as limit the duration of spatiotemporal distribution that can be assessed. Covalent linkage of a marker to the fluorophore slows release and extends the duration that can be assessed, but often results in less bright nanoparticles and may impact the particles’ surface properties. (4) The in vivo spatiotemporal distribution of nanoparticles in the eye is typically measured in timepoints rather than in real time. While biodistribution data from multiple timepoints may provide some insight into how nanoparticles distribute through the eye, its accuracy is limited by the frequency of timepoints, which is limited by the number of animals used in the study since the animal must be euthanized and the eyes collected for assessment. This encourages researchers to use cheaper and more available animal models such as rats when larger animal models such as pigs have more physiological relevance to humans.

Despite these challenges, research on ocular drug delivery is a worthy pursuit, and polymeric nanoparticles present a promising strategy for improving ocular drug delivery. To determine the optimal formulations, it is important to understand the biodistribution profiles of nanoparticles in the eye and how they are affected by nanoparticle design, administration route, and other factors. While the cornea forms a strong barrier to polymeric nanoparticles, it nevertheless can be penetrated with the aid of mucoadhesive coating and penetration enhancers. This may be the most effective approach to treat diseases of the lens, such as cataracts, as the lens lacks blood vessels and cannot be effectively reached systemically. Topically administered nanoparticles do not distribute well to the iris and ciliary body, but nanoparticles injected into the suprachoroidal space with non-Newtonian, high viscosity polymers to immobilize them is an effective approach. Systemically administered nanoparticles can also effectively reach the ciliary body and have been shown to reduce inflammation in uveitis models. Topically administered nanoparticles tend to favor conjunctival absorption, which allows them to enter the sclera and diffuse to multiple eye structures including the retina; however, elimination by entering systemic circulation remains an issue. Subconjunctival injection of nanoparticles suspended in a thermosensitive hydrogel can be used to reduce systemic clearance in the conjunctiva and enhance absorption. Subconjunctivally injected nanoparticles have been shown to reduce corneal angiogenesis and enhance corneal graft survival in animal models when used to delivery corticosteroids or plasmids containing an anti-VEGF gene. Intravitreal delivery is the most direct way to reach the retina; however, positively charged particles and particles larger than 500 nm will not effectively diffuse. Thus, smaller neutral or negatively charged particles should be used for treating retinal diseases such as AMD, retinal detachment, and diabetic retinopathy. There are several challenges for systemically administered nanoparticles to reach the inner eye, but using PEG to create a “stealth” coating on nanoparticles can help ensure successful transit, and surface ligands that bind retinal endothelial cells may improve transport across the BRB. Additional approaches should be considered when designing a nanoparticle ocular delivery system, but there is a great deal of research yet to be done to complete the picture.

## Data Availability

Not Applicable.

## Abbreviations

pRNA: packaging RNA
PLGA: Poly(lactic-co-glycolic acid)
PCL: Poly(caprolactone)
PEG: Poly(ethylene glycol)
VEGF: Vascular endothelial growth factor
DSP: Dexamethasone sodium phosphate
RGD: Arginine-glycine-aspartic acid
IOP: Intraocular pressure
PLA: Poly(lactic acid)
ROS: Reactive oxygen species
EMT: Epithelial to mesenchymal transition
CFU: Colony forming units
DMSO: Dimethyl sulfoxide
RPE: Retinal pigment epithelium
AMD: Age-related macular degeneration
CNV: Choroidal neovascularization
shRNA: Short hairpin RNA
HIF-1α: Hypoxia inducible factor -1α
BRB: Blood-retinal barrier
PVA: Poly(vinyl alcohol)
EPR: Enhanced permeability and retention
SOD1: Superoxide dismutase
Cx43MP: Connexin43 mimetic peptide
